# Applying Care Coordination Principles to Reduce Cardiovascular Disease Risk Factors in People With Serious Mental Illness: A Case Study Approach

**DOI:** 10.3389/fpsyt.2021.742169

**Published:** 2021-12-22

**Authors:** Karly A. Murphy, Arlene Dalcin, Emma E. McGinty, Stacy Goldsholl, Ann Heller, Gail L. Daumit

**Affiliations:** ^1^Division of General Internal Medicine, Department of Medicine, Johns Hopkins University School of Medicine, Baltimore, MD, United States; ^2^Welch Center for Prevention, Epidemiology, and Clinical Research, Johns Hopkins University School of Medicine, Baltimore, MD, United States; ^3^Department of Health Policy and Management, Johns Hopkins Bloomberg School of Public Health, Baltimore, MD, United States; ^4^Department of Mental Health, Johns Hopkins Bloomberg School of Public Health, Baltimore, MD, United States

**Keywords:** serious mental illness, care coordination, care management, cardiovascular risk, behavioral coaching

## Abstract

People with serious mental illness (SMI) have a 2–3-fold higher mortality than the general population, much of which is driven by largely preventable cardiovascular disease. One contributory factor is the disconnect between the behavioral and physical health care systems. New care models have sought to integrate physical health care into primary mental health care settings. However, few examples of successful care coordination interventions to improve health outcomes with the SMI population exist. In this paper, we examine challenges faced in coordinating care for people with SMI and explore pragmatic, multi-disciplinary strategies for overcoming these challenges used in a cardiovascular risk reduction intervention shown to be effective in a clinical trial.

## Introduction

People with serious mental illness (SMI) experience excess mortality at rates 2–3 times higher than the general population, equivalent to a loss of life of 10–20 years (1, 2). Cardiovascular disease (CVD) is the leading cause of death for this group and is largely modifiable by addressing risk factors, including obesity, diabetes, hypertension, dyslipidemia, tobacco use, poor diet, and physical inactivity- which are highly prevalent in populations with SMI (1, 3, 4). Use of antipsychotic medications also contributes to metabolic changes with weight gain, hyperglycemia, and dyslipidemia (5).

People with SMI often face significant challenges accessing quality healthcare, including high rates of poverty, housing instability, unemployment, and interactions with the criminal justice system (6–8). Moreover, people with SMI are less likely to receive guideline-concordant care compared with the general population (9–11). For example, they are less likely to receive annual screenings for diabetes-related complications or recommended medications after a myocardial infarction (12–15). Cognitive dysfunction, communication challenges, and low health literacy may further impede care delivery to people with SMI (6, 16, 17). Finally, specialty mental healthcare has historically been delivered separately from physical healthcare services and created challenges in coordinating services for this vulnerable population (10, 18, 19).

Care delivery models have sought to better integrate delivery of behavioral and physical healthcare, but these models have faced challenges. Models such as Collaborative Care (20–22) or the Patient Centered Medical Home (23–25), where behavioral health is integrated into primary care settings, may have primary care providers (PCPs) who lack experience in treating individuals with SMI (24). In the Behavioral Health Home (BHH), where the coordination of care for physical healthcare is centered in the specialty mental healthcare setting (26, 27), behavioral health providers may not feel equipped to address CVD risk factors (28).

Given the lower rates of guideline-concordant care and persistent mortality gap for people with SMI, it is critical to understand successful examples of care coordination around physical health conditions. Yet descriptions are scarce regarding populations with SMI. In this paper, we use a clinical vignette of an individual with SMI, who was enrolled in a successful cardiovascular risk reduction intervention trial, to highlight challenges and opportunities in delivering guideline-concordant care for people with SMI and multiple CVD risk factors. We describe the care management and care coordination processes employed within the clinical intervention and future implementation lessons.

## Care Management and Care Coordination

Care management and care coordination are complementary approaches to deliver healthcare across multiple providers and settings (Table 1). Care management is a team-based practice approach to align and manage health services according to a population's needs (29, 30). Strategies target providers (e.g., health risk assessment training, electronic decision support) or patients (e.g., health coaching, brochures) and involve a multi-disciplinary team of providers. Meanwhile, care coordination organizes patient care activities across participants involved with a patient's care (e.g., providers, patient, supporters of patient) (31). Activities include establishing accountability, communicating and sharing knowledge, facilitating transitions of care, assessing the patient's needs and goals, developing a care plan, monitoring and follow-up care, supporting patients' self-management goals, linking to community resources, and aligning resources with patient and population needs. This model has been shown to improve chronic disease care quality for the general population (29, 32, 33) and for those with SMI (34–38).

**Table 1:** Care management and care coordination activities from the Agency for Healthcare Research and Quality frameworks (30, 31).

Care management	Prioritize one-on-one encounters
	Conduct training
	Involve physicians
	Involve informal caregivers
	Provide health coaching and discrete self-management skills
Care coordination	Assess patient needs and goals
	Create proactive care plan
	Support patients' self-management goals
	Monitor and follow-up as patient's needs change
	Establish accountability
	Communicate and share knowledge
	Help with transitions of care
	Link to community resources
	Align resources with patients' needs

## Cardiovascular Risk Reduction Intervention: Ideal Trial

The NHLBI-funded IDEAL trial was a successful 18-month randomized clinical trial that tested a comprehensive cardiovascular risk reduction program incorporating care management, with an emphasis on health behavior coaching, and care coordination at four community mental health outpatient programs for people with SMI who had at least one CVD risk factor (39). It demonstrated an overall reduction in 10 year CVD risk reduction by 13% in the intervention group compared to control (40).

The intervention's theoretical framework draws upon a bio-psychosocial approach and leverages behavioral change strategy and person-centered care in addressing physical health (39). Specifically, the 269 adult participants randomized to the intervention received a care plan tailored to their specific CVD risk factors (hypertension, diabetes, dyslipidemia, tobacco use, obesity), which was delivered jointly by a health coach and nurse. The health coach, based at the community mental health organization, conducted one-on-one sessions weekly for the first 6 months and then at least every 2 weeks thereafter. Sessions focused on individual health behaviors and collaboratively agreed upon goals. The nurse met with participants around CVD risk factor education, medication counseling, and accompanied participants to physical health visits with physicians as needed. In addition, the nurse coordinated care with physical and behavioral health providers (defined broadly as any healthcare worker including pharmacists, social workers). For participants interested in smoking cessation using pharmacotherapy, the nurse coordinated with participants' psychiatrists for varenicline, bupropion, and/or nicotine replacement therapy prescriptions (39). Both the health coach and nurse used a motivational interviewing approach and solution focused therapy techniques to facilitate health behavior change with participants (39).

The institutional review boards at Johns Hopkins University and Sheppard Pratt Health System approved the clinical trial. The patient provided permission to be featured in this clinical vignette, and identifying initial was changed to protect privacy.

## Clinical Vignette

Ms. E., a participant in her mid-40s, had a medical and psychiatric history notable for hypertension, type 2 diabetes mellitus, tobacco smoking, obesity, and schizophrenia. Significant medications were metformin 750 mg twice daily, propranolol 10 mg as needed, clozapine 300 mg daily, haloperidol 5 mg twice daily, fluoxetine 60 mg daily, valproic acid 1,500 mg daily, clonazepam 0.5 mg three times day, and benztropine 0.5 mg daily. On study enrollment, laboratory values were remarkable for an elevated A1c of 7.8% (reference range <5.7%), total cholesterol of 257 mg/dl (reference range: 0–200 mg/dl), and low-density lipoprotein (LDL) of 151 mg/dl (reference range: 0–100 mg/dl) with an estimated 10-year risk of 9.2% of having a heart attack or stroke based on atherosclerotic cardiovascular disease risk factors. Ms. E. lived in a residential program, where staff helped to schedule transportation.

On study entry, Ms. E. met with the health coach and nurse and identified diabetes self-management and tobacco smoking cessation as her primary health goals. Together, the health coach, nurse, and Ms. E. formulated a care plan to reach these goals. She met with the health coach weekly where she expressed anxiety around quitting smoking and her worry about developing cancer. By the first month, she set a quit date and focused on smoking cessation through behavioral change and pharmacotherapy, consistent with an evidence-based approach for persons with SMI (41). The nurse then coordinated earlier receipt of varenicline for smoking cessation as Ms. E.'s regular appointment with the prescribing psychiatrist was several months away. Ms. E. took the prescribed varenicline, continued to receive behavioral counseling for smoking cessation, and was able to quit smoking for 4 weeks.

When Ms. E. began smoking again, with inconsistent use of varenicline, she and the health coach identified triggers that led to relapse (e.g., feeling angry), potential solutions, and set a new quit date. They focused on discrete strategies, such as avoiding situations where she may be offered cigarettes or taking deep breaths. Ms. E. restarted varenicline and met with the coach weekly. However, after a series of falls, a director of the mental health center asked to discontinue varenicline out of concern that varenicline contributed. The nurse met with the director to discuss that varenicline was unlikely the source of the falls and emphasize that Ms. E. was at high risk for tobacco relapse. They agreed that Ms. E. could continue the medication. When Ms. E. underwent surgery for an ankle fracture, the psychiatrist held varenicline in anticipation of other medication changes in the perioperative period. After surgery, Ms. E. elected not to continue on varenicline; she successfully refrained from smoking and met bi-weekly with the health coach. After she was hospitalized for a mental health crisis, Ms. E. and her coach reviewed triggers for smoking and strategies to address triggers. Ms. E. was successful in remaining tobacco free for over a year at study end.

Attaining glycemic control was challenging for Ms. E. She met with the health coach weekly around diabetes self-management skills and received educational materials with high readability and simple messaging, such as “Avoid sugar drinks,” or “What counts as a fruit.” Ms. E. and her coach reviewed her blood glucose logs and foods that she had eaten. She found it difficult to regulate portion size and to remember to choose healthy snacks (e.g., sugar-free candies as a replacement for cigarettes). The coach helped her to make a list of foods that elevated blood sugars. Ms. E. also met with the nurse and reviewed diabetes self-management, medications, and prepared questions in advance of visits with her PCP. At the visit, the nurse advocated for initiation of statin therapy, consistent with guideline-concordant lipid management in persons with diabetes (42). Ms. E. was started on pravastatin.

Throughout the following year, Ms. E. worked closely with the health coach to review food choices, increase physical activity levels, and consistently take medication. She continued to struggle with changing dietary habits- eating when stressed, eating large portions, and eating when not hungry. Ms. E. set personal goals of cutting out sugary beverages and decreasing foods high in carbohydrates. After she fractured her ankle, she worried about gaining weight. She and her coach reviewed diabetes self-management topics and aligned smoking cessation strategies with smart snacking choices. Ms. E. then set defined goals, such as choosing water instead of juice. The nurse also coordinated with the residential counselor to reschedule canceled medical appointments, arrange transportation, and to obtain regular blood monitoring of glucose levels and cholesterol. At her 18-month follow up, Ms. E. had improved glycemic and cholesterol control with an A1c of 6.5% and an LDL of 65 mg/dl on an increased dose of metformin (1,000 mg twice daily) and pravastatin 40 mg daily. Over the study, 65 encounters (78 encounters anticipated per study protocol) were documented for Ms. E. between the coach and the nurse, of which 10 involved coordination with providers outside of the community mental health program.

## Discussion

This vignette of an individual with SMI who successfully stopped smoking tobacco and achieved glycemic control with the assistance of a health coach and nurse was drawn from an 18-month clinical trial in a community setting. It highlights the real-world challenges and the intensity of resources needed to reduce CVD risk factors for persons with SMI.

### Assessing Patient Needs and Goals and Creation of a Care Plan

The care plan reflected the participant' health goals and CVD risk factors, and whether guideline-concordant care was due. It also included goals that the patient may not have prioritized (e.g., weight loss). Identifying the primary location of meals (e.g., residential facility, family) helped the health coach and nurse tailor nutrition-based education sessions (e.g., diabetes-focused) and took into account the patient's socioeconomic concerns.

Involving individuals with SMI in the creation and implementation of the care plan is fundamental to patient-centered care. This includes defining what is important to them and actions that they are willing or not to take. Motivational interviewing has been a successful approach for engaging people with SMI around health behavior change goals (43). Given that persons with SMI experience stigma within healthcare settings (16), it is important that their voice is heard from the onset and throughout care delivery.

### Supporting Disease Self-Management and Responding to Patient's Ongoing Needs

Ms. E. experienced physical health setbacks and a mental health crisis during the intervention. Intervention staff checked in frequently and adapted coaching sessions to her evolving needs and concerns. They recognized that the ankle fracture impacted her physical activity levels and influenced her cravings for snacks and cigarettes. During transitions, the health coach addressed arising anxieties and helped Ms. E. to set well-defined goals.

Our experience found continuous engagement between trial staff and participants increased participants' confidence with self-management skills. As people with SMI may experience cognitive dysfunction, disability and low health literacy, materials were tailored to improve readability and to account for these considerations (6, 44). Some participants benefited from repetition of topics, updates or selecting new targets of behavior changes. Health coaches and trial staff advocated on participants' behalf to address organizational-level challenges. For example, one participant noted that he walked past staff members who were smoking when he entered the mental health clinic. Trial staff then spoke with clinic leadership to move the designated smoking area away from the front entrance, which was a culture shift for employees. Trial staff also worked with residential facility managers to change purchasing habits (e.g., increase diet soda availability).

In addition, goal setting, and skill building reinforced a behavioral change strategy well-suited to people with SMI (43, 45). We found that aligning multiple health goals was effective. For Ms. E, she and the health coach discussed using sugar free gum and candies to address oral cravings for smoking cessation while being mindful of underlying diabetes. Similarly, health coaches encouraged setting a discrete food goal (e.g., additional serving of vegetables) that would parallel a physical activity goal (e.g., number of steps) for weight loss.

### Prioritizing One-on-One Encounters

This intervention had a high frequency and intensity of encounters between the participant, health coach, and nurse. Topics were reinforced over time, emphasized specific skills, and broke materials into small units. Coaches and nurse met participants where they were at, based on their psychiatric condition, cognitive skills, and behavioral change goals.

In addition, each health coach was embedded within a community mental health center and met with participants one-on-one. This approach likely improved rapport and facilitated communication between the coach and behavioral health team. Literature suggests that in-person encounters for care management are more effective than telephone encounters (46). Given the growth of telemedicine during the COVID-19 pandemic (47), programs should consider hybrid models for care management and care coordination services.

### Establishing Accountability

Care management and care coordination are inherently team-based practices, and successful teams need to have defined roles and responsibilities (48). During this intervention, accountability occurred when providers identified discrete expectations and goals, checked in regularly with one another, and closed feedback loops (31). The health coach and intervention nurse developed and implemented the care plan with the participant. The health coach led discussion on self-management skills and health behavior counseling, with regularly scheduled and *ad hoc* check-ins with participants. The nurse acted as a liaison with behavioral health providers, PCPs, medical sub-specialists, family, and residential program staff. The nurse sent an introductory letter about the intervention to PCPs and sometimes attended office visits. This approach helped to facilitate knowledge of how the nurse and health coach could enhance a participants' existing care plan. For example, the nurse measured participants' blood pressure and relayed that information to the PCP, thereby facilitating ambulatory blood pressure monitoring. Additional activities included advocating for aggressive management of CVD risk factors (e.g., statin prescription for cholesterol management) and educating caregivers about how to support individuals with SMI with health goals. Both the health coach and intervention nurse provided in-person and email updates to behavioral health staff of the progress of participants, if requested. If concerns related to social determinants of health arose (e.g., food insecurity), the health coach and/or nurse reached out to staff at the mental health center, which had existing ties with social services. This process required knowledge and understanding of who the participant was, their support network and living situation. Future work will need to incorporate clear expectations for roles and responsibilities and opportunities for formal and informal check-ins with team members.

### Communicating and Sharing Knowledge

When caring for people with SMI, it is essential that communication occur between the patient, behavioral health providers, and physical health providers and that communication loops are closed to reduce potential miscommunications. Transitions of care, are particularly high-risk settings (49). When Ms. E. had a somatic and later a mental health-related hospital admission, the health coach and nurse tailored counseling to match her needs to help to stay consistent with her health goals (e.g., smoking cessation).

The nurse and health coach regularly communicated in-person, by phone, and email. Frequent communication occurred with behavioral and physical health providers, with more open communication correlated to improved control of CVD risk factors for participants. However, challenges around communication arose. No shared electronic health records were available between behavioral and physical health providers, a problem observed in care coordination programs across other healthcare systems (50). Intervention staff relied on phone messages or faxes for communication with external providers, sometimes leading to delays in having messages returned from providers outside of the mental health center.

## Future Directions

This vignette illustrates how care management and care coordination processes were successfully employed to reduce CVD risk factors for person with SMI within the formalized structure of an intervention. By outlining the frequent, high-intensity care coordination and care management processes required to care for patients with SMI, we filled a gap in the literature. It is imperative that health systems implement protocolized care coordination and care management processes to address CVD risk factor care for populations with SMI.

Future program implementation may wish to start in settings that care for high number of individuals with SMI. Enhanced primary care models and BHHs are natural settings because they focus on populations with SMI, and in the case for BHHs, have an existing funding through the Medicaid waiver program (27, 51). Models that integrate somatic and behavioral health across acute and outpatient settings may also be of interest given their focus on individuals with SMI to maintain overall health after hospital admission (52).

Implementation plans will need to include accountability and address communication gaps between behavioral and physical health providers. For behavioral health providers, addressing physical health concerns may feel out of scope for their usual practice; therefore, it is essential that implementation plans include connections with (a) PCPs who can lead on physical health management, and (b) care managers and health coaches who work across behavioral and physical healthcare sectors.

Finally, financing remains a challenge. Alternative payment models, such as Accountable Care Organizations or the Medicaid waiver program, could provide funding streams to support care coordination as described here (53, 54). Future policies will need to provide support for health systems to implement these processes. This vignette is a first step in highlighting the discrete, intensive care coordination and care management processes needed to care for populations with SMI and considerations for program implementation and sustainability.

## Data Availability Statement

The original contributions presented in the study are included in the article/supplementary material, further inquiries can be directed to the corresponding author/s.

## Ethics Statement

The studies involving human participants were reviewed and approved by Johns Hopkins University School of Medicine. The patients/participants provided their written informed consent to participate in this study. Written informed consent was obtained from the individual(s) for the publication of any potentially identifiable images or data included in this article.

## Author Contributions

KM and GD contributed to the conception and design. KM wrote the first draft of the manuscript. All authors contributed to the manuscript revision, read, and approved the submitted version.

## Conflict of Interest

The authors declare that the research was conducted in the absence of any commercial or financial relationships that could be construed as a potential conflict of interest.

## Publisher's Note

All claims expressed in this article are solely those of the authors and do not necessarily represent those of their affiliated organizations, or those of the publisher, the editors and the reviewers. Any product that may be evaluated in this article, or claim that may be made by its manufacturer, is not guaranteed or endorsed by the publisher.
